# Emerging Mechanisms and Targeted Therapy of Pyroptosis in Central Nervous System Trauma

**DOI:** 10.3389/fcell.2022.832114

**Published:** 2022-03-25

**Authors:** Biao Yang, Weijie Zhong, Ying Gu, Yi Li

**Affiliations:** ^1^ Department of Neurosurgery, Ninth People’s Hospital Affiliated to Shanghai Jiao Tong University School of Medicine, Shanghai, China; ^2^ Department of Neurosurgery, Huashan Hospital, Fudan University, Shanghai, China; ^3^ Department of Neurology, The Fourth Affiliated Hospital of China Medical University, Shenyang, China

**Keywords:** pyroptosis, traumatic brain injury, spinal cord injury, target, therapy

## Abstract

Cell death can occur in different modes, ferroptosis, pyroptosis, apoptosis, and necroptosis. Recent studies have shown that pyroptosis can be effectively regulated and that like necroptosis, pyroptosis has been regarded as a type of programmed cell death. The mechanism of its occurrence can be divided into canonical inflammasome-induced pyroptosis and noncanonical inflammasome-induced pyroptosis. In the past research, pyroptosis has been shown to be closely related to various diseases, such as tumors, neurodegenerative diseases, and central nervous system trauma, and studies have pointed out that in central nervous system trauma, pyroptosis is activated. Furthermore, these studies have shown that the inhibition of pyroptosis can play a role in protecting nerve function. In this review, we summarized the mechanisms of pyroptosis, introduce treatment strategies for targeted pyroptosis in central nervous system trauma, and proposed some issues of targeted pyroptosis in the treatment of central nervous system injury.

## 1 Introduction

Traumatic central nervous system (CNS) injury such as traumatic brain injury (TBI) and spinal cord injury (SCI) can have devastating and lasting consequences for the body that result in the need for substantial medical attention throughout a patient’s life (Fouad et al., 2021). These injuries cause severe motor and neurological dysfunction and dramatically reduces quality of life and severely increases the burden on society (Burns and O'Connell, 2012). In 2016, the global incidence of new cases for TBI 2016 was 27.08 million and for SCI was 0.93 million (GBD 2016 Neurology Collaborators, 2019). Recently, in the United States alone, research has found that TBI and SCI incidences are 333 and 26 per 100,000 patients per year, respectively (Hu et al., 2020), and various studies have shown similar rates in both developing countries and developed countries (Zhou et al., 2016). Currently, methylprednisolone, surgical decompression, supportive medicine, and rehabilitation are the most common treatments for CNS trauma. However, the therapeutic effect from these treatments is not generally significant (Varma et al., 2013; Ahuja et al., 2016). Therefore, clinicians and researchers alike still seek new treatments and to understand better pathogenesis of CNS trauma.

The pathophysiological characteristics of TBI and SCI are similar, and the tissue damage caused by CNS trauma can be divided into two stages (Lipinski et al., 2015). The first stage is direct injury to the spinal cord or brain tissue caused by an external force, resulting in rupture of the cell membrane and leading to irreversible cell damage and tissue necrosis (Mortezaee et al., 2018). The second is primarily cause by some highly pro-inflammatory substance released after cell necrosis, such as glutamate, ROS, and potassium (Dixon, 2017). After this, neuroinflammation can trigger a series of secondary injuries, eventually leading to the death of neurons (Simon et al., 2017). Therefore, cell death and the subsequent inflammatory response play an important role in the long-term effects of CNS injuries (Bao et al., 2019). However, cell death itself is an essential phenomenon for maintaining the body and is critical for internal environmental homeostasis (Bogdan et al., 2016). Recently, researchers have identified the detailed mechanisms of programmed cell death (Jia et al., 2019), and evidences suggests that programmed cell death plays a critical role in CNS trauma in particular (Hu et al., 2020).

The main types of programmed cell death includes apoptosis, necroptosis, ferroptosis, autophagy, and pyroptosis (Dhanasekaran and Reddy, 2017). Apoptosis is a classical form of programmed cell death that results in the well-organized and efficient elimination of damaged cells, and apoptotic cells show characteristic cytoplasmic cell shrinkage, budding of the plasma membrane, membrane exposure of phosphatidylserine on the extracellular side, DNA fragmentation, and chromatin condensation (Pistritto et al., 2016). For necroptosis, in various pathophysiological conditions, including viral infection, cancer, and TBI, this form of regulated cell death that critically depends on RIPK3 and MLKL and generally manifests with morphological features of necrosis (Galluzzi et al., 2017). Ferroptosis is characterized morphologically by the presence of smaller-than-average mitochondria with condensed mitochondrial membrane densities, the vanishing or reduction of mitochondria crista, and outer mitochondrial membrane rupture, which can be induced by various drugs and compounds in normal and diseased tissues and is closely associated with tumor growth, neurodegenerative diseases, autoimmune diseases, and brain injury (Xie et al., 2016). Autophagy is defined as a catabolic process that is conserved among all eukaryotic organisms and plays an immensely significant role in maintaining cellular homeostasis (Saha et al., 2018). Finally, pyroptosis is a recently recognized form of programmed cell death and characterized by pore formation in the plasma membrane mediated by the gasdermin protein family (Dhanasekaran and Reddy, 2017; Broz et al., 2020). This form of programmed cell death permits the release of cellular contents, including DAMPs and inflammatory cytokines such as IL-18, which trigger the occurrence of inflammation (Frank and Vince, 2019).

Since the discovery of pyroptosis, a host of literature has studied on the mechanisms and function of pyroptosis. The relationship between pyroptosis and CNS trauma is especially worthy of further study and will surely become a hot topic in future medical research (Hu et al., 2020). As a newly-discovered form of programmed necrosis, pyroptosis is regarded as a common effector mechanism of innate immunity in vertebrates that depends on pattern recognition receptors (PRRs) to examine conserved microbial products or endogenous dangers (Jorgensen and Miao, 2015). In this review, we briefly summarize recent advances in pyroptosis research and introduce the mechanisms of pyroptosis and its regulation that may be used to create novel neuroprotective treatments.

## 2 Biology of Pyroptosis

### 2.1 The History of Pyroptosis’s Discovery and Research Developments

In 1992, some researchers found that *Shigella flexneri* or *Salmonella* infection of mouse macrophages or human monocytes resulted in cell “apoptosis”, in fact, this was not apoptosis but a new form of programmed cell death (Zychlinsky et al., 1992). In 1997, Thirumalai et al. discovered that *Shigella dysenteriae* could activate caspase-1 in human monocyte-derived macrophages and release mature IL-1β (Hilbi et al., 1997), and in 2001, two labs found that the macrophage death caused by *bacterial* infection was a mode of death that was different from apoptosis and defined it as caspase-1-dependent programmed necrosis (Boise and Collins, 2001; Cookson and Brennan, 2001). The term pyroptosis combines the Greek roots ‘pyro’ and ‘ptosis,’ which mean fever and falling, respectively, to define a newly discovered inflammatory programmed cell death (Shi et al., 2015). In 2017, Shi et al. (2017) officially named pyroptosis as a form of gasdermin-mediated programmed necrotic cell death.

For a long time, pyroptosis had been defined as caspase-1-mediated monocyte death in response to toxin-stimulated or pathogen-infected macrophages (Bergsbaken et al., 2009). Recently, however, a critical study found that after the cleavage and activation of caspases-1/4/5/11, gasdermin D (GSDMD) was divided into two fragments: N-terminal gasdermin D (GSDMD-NT) and C-terminal gasdermin D (GSDMD-CT) (Shi et al., 2015). The laboratory of Feng Shao found that proteins in the gasdermin family such as GSDMD played an important role in membrane pore-forming activity in response to specific bacterial attacks, and they defined pyroptosis as gasdermin family-mediated programmed necrosis, which aroused much attention in the cell death and immunity research community (Shi et al., 2017). Moreover, the Nomenclature Committee on Cell Death (NCCD) in 2018 updated defined pyroptosis as a form of regulated cell death that critically relies on the formation of plasma membrane pores by proteins of the gasdermin family, often (but not always) as a consequence of inflammatory caspase activation (Galluzzi et al., 2018). Members of the gasdermin family (including GSDMB/C/D/E) have been found to be cleaved by caspases and the gasdermin N-terminal to be used to form pores, leading to pyroptosis (Liu X et al., 2021)*.* Researches related to the primary history of the discovery of pyroptosis are shown in Table 1.

**TABLE 1:** The history of the discovery of pyroptosis and its related research developments.

Year	Stimulators	Pathway	Results	References
1992	*Shigella flexneri*	—	Apoptosis in infected macrophages	Zychlinsky et al. (1992)
1997	*Shigella dysenteriae*		Activate caspase-1 in host cells	Hilbi et al. (1997)
1999	*Salmonella*	Caspase-1	The cell death	Hersh et al. (1999)
2001	—	—	A programmed cell death occurs through caspase-1 dependent mechanism	Boise and Collins (2001)
2015	Caspases 1/4/5/11	GSDMD	Pyroptotic cell death	Shi et al. (2015)
2017	Chemotherapy drug	Caspase-3/GSDME	apoptosis	Wang et al. (2017)
2018	Caspase-8	GSDMD	Pyroptosis	Sarhan et al. (2018)
2020	GSDME	CAR T cell	Cytokine release syndrome	Liu Y et al. (2020)
2020	GAZA	GSDMB	Pyroptosis	Zhou et al. (2020)
2020	Caspase-8	GSDMC	Pyroptosis in cancer cells	Hou et al. (2020)

### 2.2 The Molecular Characteristics of Pyroptosis

#### 2.2.1 The Cleavage and Activation of Gasdermins

Caspases are a family of intracellular cysteine proteases and play an essential role in cellular demise (Franchi et al., 2009). However, several caspases such as the human caspase-1, -4, and -5 and the murine caspase-1, -11, and -12 are related to the activation of pro-inflammay molecules, and are therefore referred to as “pro-inflammatory caspases” (Martinon and Tschopp, 2007). Active caspase-1 cleaves GSDMD, pro-IL-1β, and pro-IL-18 into their mature, biologically active forms; GSDMD-NT participates in pyroptosis, and the IL-1β/18 has been implicated in multiple immune reactions (Arend et al., 2008). Caspase-11 is a murine caspase molecule, which is 60% similar to the human caspase-4/5 sequence, and its activation pattern of pyroptosis is also similar to that of humans (Kayagaki et al., 2011). Caspase-11 is a receptor of intracellular lipopolysaccharide (LPS), and recognizes and binds LPS to activate the formation of the non-classical inflammasome and induces cell scortosis without the involvement of caspase-1, which is different from Caspase-1 (Shi et al., 2014)*.* However, activation of caspase-1 results in the cleavage and secretion of IL-1β and IL-18 (Kayagaki et al., 2011). Activated caspase-11 activates NLRP3 inflammasome and then processes the cleavage and activation of IL-1β and IL-18 by caspase-1 (Kayagaki et al., 2011). Finally, caspase-3 cleaves GSDME into GSDME-NT and GSDME-NT and can penetrate the cell membrane to form pores (Liu Y et al., 2020).

#### 2.2.2 The Gasdermins are the Executors of the Pyroptosis

The members of the gasdermin protein family are the executors and substrates of pyroptosis (Broz et al., 2020). The Feng Shao group defined pyroptosis as gasdermin family-mediated programmed cell death (Shi et al., 2017). The gasdermin family in human contains six members, GSDMA (also known as GSDM, GSDM1, or FKSG9), GSDMB (also known as GSDML, PP4052, or PRO2521), GSDMC (also known as MLZE), GSDMD (also known as GSDMDC1, DFNA5L, or FKSG10), GSDME (also known as ICERE-1 or DFNA5), and PJVK (also known as DFNB59) (Broz et al., 2020). Among them, GSDME and DFNB59 are the most evolutionarily ancient members of gasdermin family (Kersey et al., 2018), and gene sequences of GSDMA are found in mammals, reptiles, and birds. However, GSDMB, GSDMC, and GSDMD genes are exclusively present in mammals and are closely associated with GSDMA, suggesting that they arose through gene duplication (Broz et al., 2020).

Structurally, each member of the gasdermin family consists of two different domains connected by a flexible linker, except for PJVK, which only contains a smaller C-terminal part (Broz et al., 2020). The gasdermin N-terminal (GSDM-NT) domain shows the highest similarity in gene sequence among the gasdermin members. In contrast, the gasdermin C-terminal (GSDM-CT) domain presents lower sequence similarity (Kayagaki et al., 2015). More importantly, many studies have shown that GSDM-NT is responsible for the pore-forming of the cell membrane and the activity of pyroptosis, although GSDM-CT can suppress the activity of GSDM-NT *via* binding with GSDM-NT (Kayagaki et al., 2015).

Activation of caspase-1/11 in mice and caspase-1/4 in humans has been reported to cleave GSDMD at its central linker region (LLSD in the mice and FLTD in humans) to produce two fragments: GSDMD-NT and GSDMD-CT (Shi et al., 2015), but only GSDMD-NT can induce membrane permeabilization and pyroptosis via pore-forming of the cell membrane, allowing the releases of IL-1β and IL-18. In addition, GSDMD-CT acts as a suppressor by linking to GSDMD-NT (Kayagaki et al., 2015; Shi et al., 2015). Researchers have also found that GSDMB was is highly expressed in specific tissues but that it appears to be silenced in tumors such as those caused by gastric and esophageal cancers. Moreover, the Cancer Genome Atlas (TCGA) database shows a strong positive association between the expression level of GSDMB and overall survival of bladder carcinoma and skin cutaneous melanoma patients (Zhou et al., 2020). Cytotoxic lymphocyte-mediated immunity depends on the release of granzymes. A recent study reported that GSDMB was cleaved into GSDMB-NT and GSDMB-CT by lymphocyte-derived GZMA from natural killer cells and cytotoxic T lymphocytes, which released its pore-forming activity of GSDMB-NT and that IFN-γ improved the expression level of GSDMB and further promoted the occurrence of pyroptosis (Zhou et al., 2020). Other recent studies reported that human and mouse GSDME was cleaved by caspase-3 in their linker region to cause pyroptosis and loss of membrane integrity after cells have already entered into apoptosis (Shi et al., 2015; Wang et al., 2017).

## 3 The Mechanisms of Pyroptosis

For a long time, pyroptosis was deemed to be a caspase 1-dependent death in response to specific bacterial attacks (Shi et al., 2015). After a long development, however, studies found that pyroptosis was the primary effector mechanism of pro-inflammatory caspases, a group of proteases activated within the inflammasome complex (Broz and Dixit, 2016). Two different signaling pathways, including the canonical and non-canonical inflammasome pathways, sense pathogen-derived or host-derived danger signals and initiate the activation of caspase-1/11 in mice or caspase-1/4 in humans (Broz et al., 2020).

### 3.1 Canonical Inflammasome-Induced Pyroptosis

**FIGURE 1:**
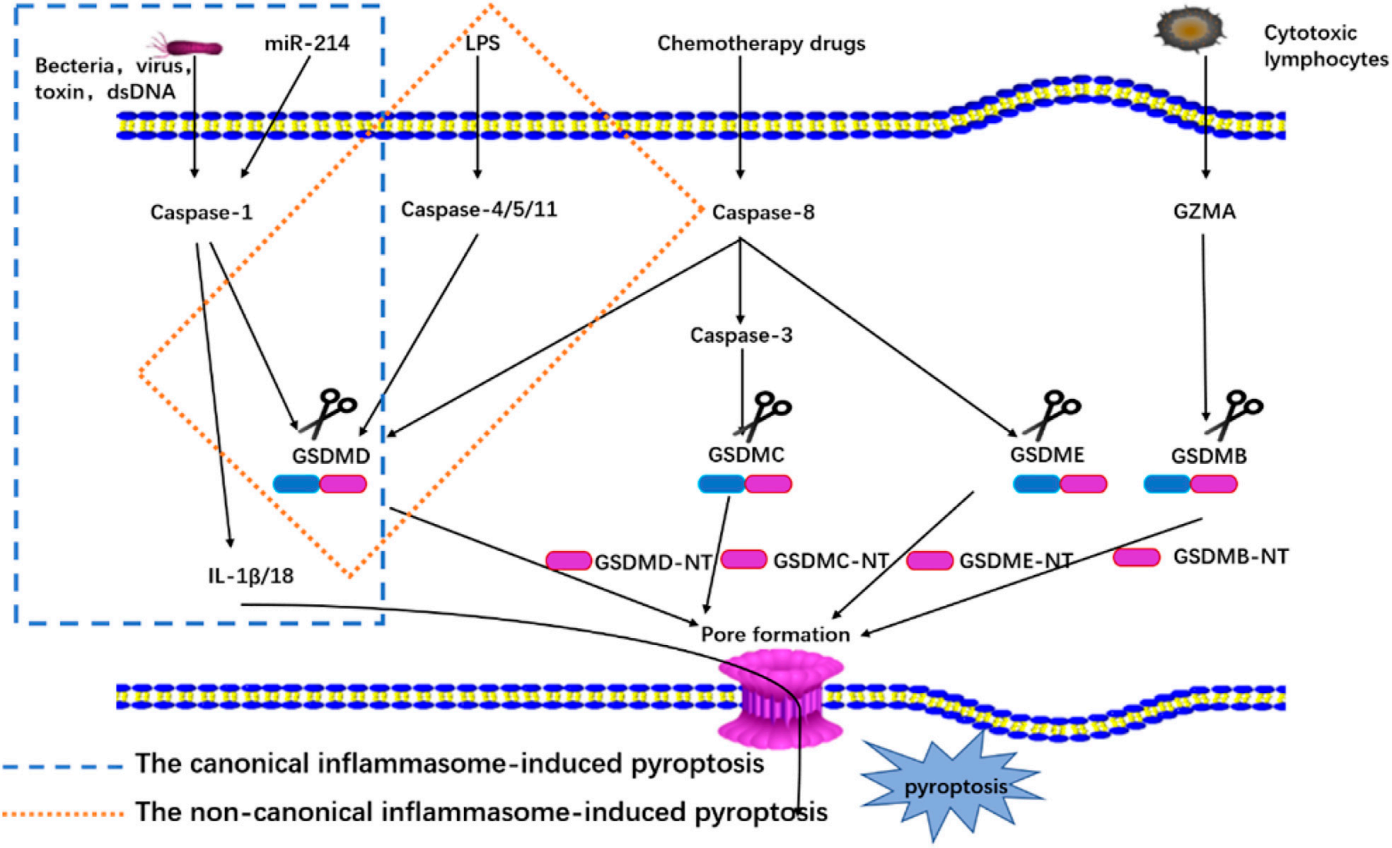
Mechanisms of pyroptosis.

The canonical inflammasome complex has been reported to play an essential role in innate immunity and as a molecular platform that triggers the activation of caspase-1 and the processing of pro-IL-1β (Martinon et al., 2002). The canonical inflammasome complex consists of the nucleotide-binding oligomerization domain (NOD)-like receptor (NLR) family (NLRP1, NLRP3, and NLRC4), absent in melanoma 2 (AIM2), or pyrin proteins through the action of pathogen-associated molecular patterns (PAMPs) and danger-associated molecular patterns (DAMPs), and it is activated by alteration to internal homeostasis or endogenous danger signals (Kayagaki et al., 2011; Broz and Dixit, 2016). The receptors collect apoptosis-associated speck-like adaptor protein that contains a caspase recruitment domain (ASC) to process pro-caspase-1 via the interactions of pyrin domains (PYD) or caspase activation and recruitment domains (CARD) (Shi et al., 2014). Then, caspase-1 is activated by the canonical inflammasome complex, and mature caspase-1 cleaves GSDMD into GSDMD-NT and processes pro-IL-1β and pro-IL-18 into mature cytokines (Shi et al., 2014). Based on the membrane pore-forming activity of GSDMD-NT, pyroptosis is thus achieved, and mature IL-1β and IL-18 are released (Aachoui et al., 2013). This pathway is shown in Figure 1.

### 3.2 Non-Canonical Inflammasome-Induced Pyroptosis

The non-canonical inflammasome pathway has been induced by activating pro-caspase-11 in mice (pro-caspase-4/5 in humans) (Shi et al., 2017). Here lipopolysaccharide (LPS) from Gram-negative bacteria is identified and bound to promote the oligomerization and activation of pro-caspase-11 in mice (pro-caspase-4/5 in humans). Then, the caspases are processed into mature caspase (Aglietti et al., 2016; Shi et al., 2017). Next, caspase-4/5/11 directly cleaves GSDMD into two fragments, GSDMD-NT and GSDMD-CT, and the GSDMD-NT fragment can forms pores on the cell membrane (Aglietti et al., 2016). The pores result in the activation of the NLRP3 inflammasome and the maturation and release of IL-1β/IL-18, and notably, caspase-4/5/11 does not directly process pro-IL-1β/18 (Kayagaki et al., 2011). This pathway is shown in Figure 1.

### 3.3 Pyroptosis Induced by Other Molecules

Many studies have also discovered that some novel molecules can also participate in pyroptosis. One recent study reported that miR-214 can recognize caspase-1 and inhibit cell proliferation and migration through the regulation of pyroptosis intermediated by caspase one in glioma cells (U87 and T98G) (Jiang Z et al., 2017), and another study showed that CAR T cell can release GZMB and then process pro-caspase-3 into mature caspase-3 in target cells to cleave GSDME, producing the N-terminal fragment of GSDME, which promotes Finalymembrane pore formation to induce pyroptosis (Liu Y et al., 2020). Additionally, many other studies have reported that chemotherapy drugs also can also cleave the pro-caspase-3 involved in pyroptosis in both cancer and normal cells with a high expression level of GSDME (Rogers et al., 2017; Wang et al., 2017). Other research has found that GSDMB is cleaved into two fragments by lymphocyte-derived GZMA from immune cells, which releases its pore-forming GSDMB-NT and that IFN-γ can improve the expression level of GSDMB and further induce pyroptosis (Zhou et al., 2020). Finally, under the stimulation of TNF-α, caspase-8 specifically cleaved GSDMC to produce GSDMC-NT, and forms pores in the membrane to cause pyroptosis (Hou et al., 2020)*.* The pathway is shown in Figure 1.

## 4 Pyroptosis Inhibitors

Pyroptosis is believed to be a key factor that results in many pathological conditions, and most of our knowledge about pyroptosis has been gained through the use of pyroptosis inhibitors, which can be classified according to different their targets of action.

### 4.1 Caspase-1 Inhibitors

Previous studies have indicated that caspase-1 is a critical component to pyroptosis, and that when inhibited it can attenuate pyroptosis in disease (Hu et al., 2020), including in CNS injury. This provides a theoretical basis for using caspase-1 inhibitors as therapeutic agents. VX-765, a selective small molecule, can block caspase-1 activation by covalently modifying the catalytic site of caspase-1 and thusly inhibit caspase-1 dependent pyrotposis (Cornelis et al., 2007; Audia et al., 2018). Similarly, ac-YVAD-cmk is also known as a selective inhibitor of caspase-1 (Lin et al., 2018). It exerts a neuroprotective effect by inhibiting caspase-1-dependent pyroptosis in both ischemic stroke and TBI model (Hara et al., 1997).

### 4.2 NLRP3 Inhibitors

Previous research has shown that NLRP3 signaling pathways are a crucial step in pyroptosis. Thus, they have been considered a target for regulating pyroptosis (Pétrilli et al., 2007). MCC950, a specific small-molecule inhibitor, was first identified as an IL-1β inhibitor (Perregaux et al., 2001), and today, it has become as an NLRP3 inflammasome inhibitor (Wu et al., 2020). Interestingly, MCC950 attenuates caspase-1 and IL-1β secretion (Zhang et al., 2017) and affects the assembly steps of NLRP3 inflammasome, but not the initiation step of NLRP3 inflammasome activation (Coll et al., 2015). CY09, identified by Jiang H et al. (2017), is regarded as a the cystic fibrosis transmembrane conductance regulator (CFTR) channel inhibitor. Different from MCC950, CY09 inhibits NLRP3 by directly binding to the Walker A motif of NLRP3, terminating the ATP binding of NLRP3 to inhibit its ATPase activity (Yang Y et al., 2019). Similarly, an active β-sulfonyl nitrile called OLT1177, Marchetti et al. (2018) has an anti-inflammatory effect on NLRP3 inflammasome and according to inhibits ATPase activity. Additionally, there are some other NLRP3 pathways still under study, such as Tranilast (Huang et al., 2018), Oridonin (Yang H et al., 2019), and glyburide (Shao H et al., 2020).

### 4.3 GSDME Inhibitors

GSDMD inhibitors are still poorly understood (Ruan et al., 2020). Necrosulfonamide (NSA), a small molecule, is known as a potent inhibitor of mixed lineage kinase domain-like protein (MLKL) (Sun et al., 2012), and Rathkey et al. have found that it can also inhibit pyroptosis (Rathkey et al., 2018). Thus, NSA is not a selective inhibitor for GSDMD. Bay 11-7082, however, was first identified as an inhibitor of the NF-κB and NLRP3 inflammasome pathways (Irrera et al., 2017). Interestingly, it also exerts an inhibitory effect on pyroptosis through GSDMD. In addition, a biorthogonal system to GSDMA3 indicated that pyroptosis in tumor cells possibly enhanced the antitumor immune response and heightened the efficacy of immune checkpoint blockade (Wang et al., 2020). Whether these drugs can be used to treat CNS injury remains to be investigated, however.

### 4.4 Other Inhibitors

In addition, other than those discussed have also been used to regulate pyroptosis. Studies have shown that the cathepsinB (CTSB) inhibitor CA-074Me can inhibit silica and asbestos-induced NLRP3 inflammasome activation (Hentze et al., 2003), and A438079, an experimental agent that inhibits P2X7R activity, not only reduces the expression of ASC, but also inhibits the NF-κB pathway (Yan et al., 2015). Recent experiments have found that salidroside, a new experimental agent, can inhibit pyroptosis by inhibiting the expression of inflammatory cytokines and NF-κB and MAPK signaling pathways (Su et al., 2019)**.**

## 5 The Role of Pyroptosis in CNS Trauma

In existing studies, scholars have found that pyroptosis is closely related to neurological function and brain injury caused by CNS trauma through both *in vivo* and *in vitro* experiments. Furthermore, the regulation of pyroptosis-related proteins, such as NLRP3 and caspase-1, can alleviate the damage caused by CNS trauma (Table 2).

**TABLE 2:** The role of pyroptosis in CNS trauma.

Disease	Intervention	Target	Function and mechanism	Reference
TBI	MCC950; JC124; Gene knockout	NLRP3	Reduce protein expression levels of NLRP3, ASC, IL-1 beta, inhibit pyrolysis, and reduce brain damage	Kuwar et al. (2019), Ismael et al. (2018)
	CORM-3	NLRP3/GSDMD/Cleaved caspase-1	Effectively resist neuroinflammation, inhibit pyroptosis, and improve prognosis	Zhang et al. (2021)
	Rhein	Caspase-1/GSDMD/IL-1β/IL-18	Inhibit TBI-induced neuronal pyrolysis to alleviate neurological deficits.	Bi et al. (2020)
	siRNA knockdown and VX-765	Caspase-1	Ablation of caspase-1 can inhibit TBI-induced pyrolysis and nerve function damage	Liu et al. (2018)
	Ac-YVAD-cmk	Caspase-1; NLRs and AIM2 inflammasome	Inhibition of caspase-1 blocks the assembly of NLRs and AIM2 inflammasomes, thereby inhibiting pyrolysis	Ge et al. (2018)
SCI	BAY87-2243; MK2206	HIF-1α inhibitor; AKT inhibitor	CD73 exerts an anti-pyroptosis effect in SCI through PI3K-AKT-Foxo1 signaling pathway	Xu et al. (2021)
	A438079	P2X7R and Caspase-1	Inhibition of P2X7R activation further inhibits caspase-1 activation and promotes the functional recovery of mice after SCI	Jiang W et al. (2017), Wang et al. (2015)
	Celastrol; kaempferol	Inflammation and pyroptosis	Celastrol and kaempferol exerts a neuroprotective effect by inhibiting the inflammation caused by microglia pyrolysis and excessive activation after SCI	Dai et al. (2019), Liu Z et al. (2021)
	Wogonoside	NF-kB/TLR4	Reduces neuronal loss and pyroptosis; Improves functional recovery.	Zhu et al. (2017)
	Quercetin	NLRP3/IL-1b/IL-18	Inhibit pyroptosis, promote neuroprotective axon regeneration, and promote neurological rehabilitation	Jiang et al. (2016)

### 5.1 Pyroptosis and TBI

#### 5.1.1 *In Vitro*

In embryonic cortical neurons, Adamczak et al. activated the AIM2 inflammasome and induced cell death. They then observed the activation of caspase-1 and secretion of IL-1β. After that, they observed that probenecid, a non-selective pannexin1 channel inhibitor (Wang et al., 2013), can inhibit neuronal pyroptosis (Adamczak et al., 2014). Numerous pieces of evidence indicate that inflammasomes are involved in pyroptosis-induced by TBI injury (Mortezaee et al., 2018). Liu et al. (2018) set up an injury model on the primary neurons. Interestingly, they found that neuron injury induced not only apoptosis but also pyroptosis and neuroinflammation. They detected the higher expression level of caspase-1 and GSDMD as well. After utilizing caspase-1 siRNA and VX-765 to inhibit the expression of caspase-1, the neuroinflammation was significantly reduced. The Zeng et al. (2014), and using the same model as Liu et al., Ma et al. detected the expression of inflammation factors and pyroptosis-related proteins. All their results indicated that Rhein treatment was able to ameliorate neuron injury induced by pyroptosis and inflammation (Bi et al., 2020).

#### 5.1.2 *In Vivo*

TBI can induce neurological deficits (Dang et al., 2017), and as previously mentioned pyroptosis will be induced after TBI. Liu et al. (2018) proposed that the knockout of caspase-1 can drive the anti-inflammatory response of the damaged cortex and inhibit the cell pyroptosis induced by the acute phase of TBI. Similar to this research, Sun et al. (2020) indicated that VX765, a selective caspase-1 inhibitor, exerts a neuroprotective effect after TBI. Moreover, they demonstrated that inhibition of VX765 can inhibit the inflammatory response of nerves after TBI. In addition and similar to their own research mentioned above, Ma et al. used Rhein to intervene in a TBI mouse model and found that Rhein can protect against nerve function damage after TBI by inhibiting pyrolysis (Bi et al., 2020). Gao et al. (2020) investigated the relationship between autophagy and pyroptosis by activating autophagy in a mouse model of TBI and observed that autophagy further inhibited pyroptosis after TBI by down-regulating IL-13 and inhibiting the JAK-1 signaling pathway, thereby exerting a neuroprotective function. Taking a different approach, Ge et al. (2018) proposed that pyroptosis mediated by NLRs and AIM2 inflammasomes is an essential factor in the aggravation of blood-brain barrier damage after TBI, and that targeted inflammasome treatment of TBI can help restore its nerve function. Additionally, in a TBI model of rats, treatment of TBI rats with JC124, a specific NLRP3 inflammasome inhibitor, has been found to reduce the number of degenerated neurons and the area of lesions caused by injury significantly. These protective effects are all through the inhibition of pyrolysis-related proteins such as NLRP3, caspase-1, and IL-1β (Kuwar et al., 2019). Interestingly, HIF-1α can also activate NLRP3-mediated pyrolysis and aggravate TBI, and the use of HIF-1α inhibitors (LW6) can reverse the effect of HIF-1α on TBI (Yuan et al., 2021).

TBI not only causes neurological deficits but also causes a series of complications, such as acute lung injury (ALI), acute respiratory distress syndrome (ARDS), and anxiety and depression-like symptoms (van der Naalt et al., 2017; Hanna et al., 2020). CORM-3 is an exogenous CO donor. In a TBI model on rats, CORM-3, which is an exogenous CO donor, treatment mitigated neurological dysfunctions via reduced neuronal pyroptosis (Zhang et al., 2021), and CORM-3 can also alleviate mental disorders after TBI by activating PKG-ERK1/2 signaling and inhibiting pyroptosis (Li Y et al., 2020). Ghrelin, a 28-amino acid peptide secreted primarily in the stomach, is also known for its protective effect in neuro damage (Qi et al., 2014), and Shao XH et al. (2020) found that Ghrelin has a protective effect on ALI after TBI. These effects occur by blocking NF-κB signaling to improve inflammasome-mediated pyroptosis.

### 5.2 Pyroptosis and SCI

#### 5.2.1 *In Vitro*

Many studies have examined SCI-related pyroptosis. One in particular, used the natural anti-inflammatory compound in a model of microglial cells (Kannaiyan et al., 2011). In this research the SCI group significantly increased the M1 microglia, and this protective effect is achieved by inhibiting microglial activation and pyroptosis (Dai et al., 2019). In the same cell model, Xu et al. (2021) investigated CD73 and microglia pyroptosis. They proposed that CD73 can inhibit microglia pyrolysis and relieve neuroinflammation. More importantly, after they induced inflammation and pyroptosis on microglia through LPS, they found that CD73 can effectively alleviate microglia pyroptosis through the PI3K/AKT pathway.

In addition to TBI, CORM-3 can also significantly alleviate neuronal pyroptosis in SCI (Zheng et al., 2019). Hv1, a voltage-gated proton channel, can regulate the function of microglial (Tian et al., 2016). And in the oxygen-glucose deprivation/reoxygenation (OGD/R) of PC12 cells, Li X et al. (2020) observed that Hv1 deficiency inhibits PC12 cell pyroptosis by inhibiting ROS. In LPS-induced microglial, Xu et al. (2020) proposed TLR4 can regulate pyroptosis after SCI by lncRNAs. They observed that TLR4 was activated after SCI. Here, the use of TLR4 inhibitors in microglia was able to down-regulate the expression of lncRNA-F630028O10Rik significantly, and lncRNA-F630028O10Rik abolished the anti-pyroptosis effect of TLR4 deficiency. The authors further found that the TLR4 signaling pathway regulates lncRNA-F630028O10Rik through STAT1.

#### 5.2.2 *In Vivo*

In an SCI model of rats, AOPPs and oxidative stress levels has been found to increase significantly, and pyroptosis has been induced in BV2 cells through the nox4-ROS-NLRP3-GSDMD signaling pathway. In one study, apocynin, an NADPH oxidase inhibitor (Sparrey et al., 2016), significantly reduced oxidative stress and cell coke death after SCI and promoted motor function recovery and histological level (Liu Z et al., 2020). In the same model, celastrol can improved the motor function and severity of SCI by inhibiting the expression of pro-inflammatory factors and the occurrence of pyroptosis (Dai et al., 2019). In addition, in the SCI model of CD73 knockout mice, Xu et al. observed that the lack of CD73 promoted the activation of microglia and NLRP3 inflammasomes *in vivo*. After treatment with BAY87-2243, which is an HIF-1α inhibitor (Griggio et al., 2020), they found that HIF-1α and CD73 promote each other and jointly regulate microglia pyroptosis (Xu et al., 2021). In a SCI model of Hv1 knockout mice, pyroptosis of neurons was inhibited, and motor function and axonal regeneration were alleviated after SCI (Li X et al., 2020). Similar to this research, Xu et al. (2020) found that TLR4 deficiency can further alleviate the recovery of motor function after SCI by inhibiting microglia pyrolysis.

## 6 Prospective Research

Although the current research suggests that pyroptosis is involved in the disease progression of CNS trauma, research has also shown that intervention with essential pyroptosis-related proteins in different animal models or cells can play a crucial role in protecting neurological function. However, there is no valid evidence in clinical studies that inhibition of pyroptosis protects CNS trauma. In general, proteins such as NLR family, Caspase-1 and GSDMD play crucial roles in the process of pyroptosis. Therefore, clinicians may want to target pyroptosis-related proteins for inhibition or gene modification in clinical settings. However, although current pyroptosis inhibitors have been shown to be effective in animal models, they may not be effective in humans due to species differences. In many, many past studies, drugs have had significant preclinical efficacy, but were found to be ineffective during clinical trials. Thus, we recommend more clinical research to investigate the relationship between pyroptosis and CNS trauma.

The above-mentioned inhibitors that target pyroptosis-related proteins, such as VX765, are involved in both apoptosis and pyroptosis, but the primary mechanisms of their protective effects in CNS trauma remain unclear. Most of the studies on these inhibitors have been conducted on their protective effects from inhibiting pyroptosis-related proteins, but there are few studies on the potential toxicity of these inhibitors and whether different administration methods have an impact on the treatment efficacy. The half lethal dose, effective half rate, and dose concentration curve of these inhibitors still need to be further studied in different animal and cell models, which is crucial before clinical application.

Finally, activating autophagy can inhibit the occurrence of pyroptosis, and research has shown that in the event of CNS trauma, there is crosstalk in different ways of cell death. Exploring the relationship between different cell modes of death and pyroptosis in CNS trauma is vital to understanding them further.
